# Comparison of the Hounsfield unit in CT scan with the gray level in cone-beam CT

**DOI:** 10.15171/joddd.2019.028

**Published:** 2019-10-07

**Authors:** Tahmineh Razi, Parya Emamverdizadeh, Nadia Nilavar, Sedigheh Razi

**Affiliations:** ^1^Department of Oral and Maxillofacial Radiology, Faculty of Dentistry, Tabriz University of Medical Sciences, Tabriz, Iran; ^2^Department of Oral and Maxillofacial Pathology, Faculty of Dentistry, Tabriz University of Medical Sciences, Tabriz, Iran

**Keywords:** Computed tomography, cone-beam computed tomography, gray level, Hounsfield unit

## Abstract

***Background.*** The present study was undertaken to compare the Hounsfield Unit (HU) in computed tomography (CT) with the gray level in CBCT in human tissues.

***Methods.*** In this study, 25 different soft and hard tissues were evaluated in 21 patients. CBCT images were taken with Newtom VGi machine (Verona, Italy) and CT images were prepared with Somatom Sensation unit (Siemens, Germany). The HU values of soft and hard tissues were compared with the gray level values of CBCT images.

***Results.*** There was a strong correlation between the HU in CT and the gray level in CBCT in soft tissues (P<0.001, R^2^ = 0.85) and hard tissues (P<0.001, R^2^ = 0.74) and in general (P<0.001, R^2^ = 0.91).

***Conclusion.*** A high degree of agreement was seen between HU in CT and gray level in CBCT in both hard and soft tissues. Since the gray level in CBCT was similar to HU in CT and can be used as a parameter determine bone density in implant treatment and also to determine the bone type, the CBCT technique is recommended in such cases due to its low radiation dose, short time and low cost compared to CT.

## Introduction

Computed tomography (CT) images are used for the evaluation of soft and hard tissues and the diagnosis of pathologic and traumatic lesions in the head and neck region.^1^ CT has a standard design to measure beam attenuation by the body issues, which is referred to as Hounsfield Unit (HU). HU is used to evaluate the quality of bone at implant placement area, to control grafts and to diagnose lesions, anatomic structures, etc.^2^

With the ever-increasing use of cone-beam computed tomography (CBCT) in the maxillofacial region, evaluation of the quality of bone with this technique has drawn attention; the gray level has been used to this end. It should be taken into account that the gray level is not the same as the true HU.^3,4^ The gray level, too, can be used to determine the type of bone for placing dental implants, to evaluate the airways, to assess the stability of grafts and to diagnose some pathologic lesions.^3-6^

Some studies have shown that the CBCT technique cannot accurately show HU, which might be attributed to its high scattered radiation dose, artifacts and the noise resulting from the use of a cone-shaped beam in the CBCT, making the CBCT unreliable for estimating the density of bone. In contrast, some studies have shown a strong linear relationship between HU in CT and gray level in CBCT. For example, in a study by Katsumata et al,^7^ the gray level of bone had a wide range from -1500 to +3000, limiting the ability to evaluate the quality of bone. Mah et al^3^ introduced a technique in which HU could be derived from the gray level. They compared the HU derived from a linear correlation coefficient with that derived from the gray level and reported minor differences in the majority of cases.

In a study by Reeves et al^8^ in 2012 on humans, comparative evaluations were carried out between the CBCT systems, only with the use of artificial materials placed in the patients’ oral cavities, and attenuation by human tissues was not compared. In addition, in that study, comparisons were not carried out with the true HU of the imaging system, and the HU values were presented as values achieved from the relationship acquired from previous in vitro studies.^8^

In a study on a human sample, which was carried out on the human cadaver mandible, evaluation of the implant placement site showed that the gray level and HU values were significantly different.^4^

The phantoms used in the majority of previous studies have had a homogeneous density in the entire structure of the material.^3,5,9,10^ In addition, with the use of dry mandible in previous studies, the effect of soft tissues has been eliminated, and only hard tissues have been evaluated, which is different from the structure of the tissues in living humans.^4^ Furthermore, considering the ever-increasing clinical use of the gray level and the advantages of CBCT over CT, the present study was undertaken to compare the HU and the gray level.

## Methods

The CT scan images in the archives of Imam Reza Educational and Treatment Center, Tabriz, Iran, were used for the purpose of this study. The local Ethics Committee approved the protocol of the study (The letter number is IR.TBZMED.REC.1395.248). The patients had undergone CT scan examinations for various reasons, followed by CBCT examinations in Tabriz Faculty of Dentistry. Twenty-one patients were included in the study in terms of the inclusion and exclusion criteria, consisting of 16 males and 5 females, with an age range of 22‒70 years. The inclusion criteria consisted of patients >20 years of age, with complete formation of compact bone.

The exclusion criteria consisted of images with beam hardening caused by metal or other dense objects, patients with CT or CBCT image on which the maxillofacial region had been depicted incompletely, patients with very severe traumatic or pathologic injuries leading to the destruction of the tissues to be evaluated, patients with conditions affecting bone density such as systemic conditions and those affecting soft tissues, and those taking medications that affected the bone density and soft tissues.

The spiral CT scan examinations had been carried out with the use of Somatom Sensation CT scan unit (Siemens, Germany) with a resolution of 0.4 mm at mAs of 32 and kVp of 140, adjusted in terms of the patients’ age and gender. Syngo CT2009E software program was used for the initial and final reconstruction of the images.

CBCT examinations had been carried out with the use of NewTom VGi unit (Verona, Italy), which delivers a cone-shaped x-ray beam with a 360° rotation, 0.3-mm voxel size, and 18-second scan time at kVp=110. The machine has a flat-panel detector and a pixel size of 1920×1536 that regulates the exposure conditions automatically. NNT viewer 2.17 software program was used for the initial and final reconstruction of the images.

Both the CT and CBCT images were evaluated on a 19-inch LCD monitor (PHILPS, 190B) with a resolution of 1024×1028 and 32 bits in a windowless dimly lit room by an experienced postgraduate student and an oral and maxillofacial radiologist (with a high rate of inter-observer agreement). A slice thickness of 1 mm was selected in both units. and the images were examined at axial, coronal and sagittal cross-sections after adjusting the midline. A medical radiologist supervised the evaluation procedures.

Twenty-five different tissues were evaluated, consisting of the paranasal sinuses, lips, the tongue, soft palate, uvula, lens, ocular fluids, optic nerve, brain, cella turcica, parotid glands, masseter muscles, pulp, enamel, dentin, coronoid processes, condyles, mandibular cortical bone, mandibular cancellous bone, tuberosity spongy bone and anterior nasal spine.

**Figure 1:**
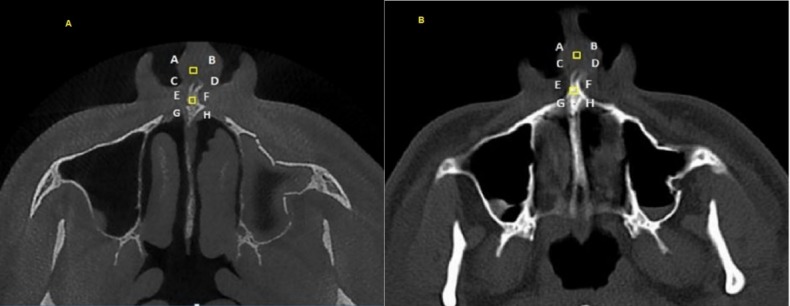
Use of the anterior nasal spine in the axial cross-section to evaluate a 2-mm square for the evaluation of nasal soft tissues at a distance of 2 m in front of it; A. CBCT view; B. CT view.

Four points were considered for each tissue and based on the anatomic landmarks in the area, they were matched in the CT and CBCT images. To this end, both images of each patient were simultaneously evaluated, and the corresponding points in that area were determined. For example, to evaluate the nasal area in the axial cross-section, squares measuring 2 mm on each side were used at a distance of 2 mm from the area in front of the anterior nasal spine (Figure 1); as another example, in order to evaluate the enamel in patients with central incisors, 4 points on one line were considered at a distance of 1 mm from the incisal edge. The alveolar crest emergence point was used on both images to evaluate the pulp in the axial cross-section.

### Statistical Analysis

Data were analyzed with descriptive statistics and regression test, using SPSS 20. Statistical significance was set at P<0.05.

## Results

In the present descriptive study, 21 human samples were evaluated, consisting of 5 females (24%) and 16 males (76%), with an age range of 22‒70 years and a mean age of 42 years.

### Analysis of Soft Tissues

Linear regression analysis was used to evaluate the relationship between CBCT and CT concerning soft tissues. Table 1 presents the results of the analysis. Based on the results, there was a strong linear relationship between CBCT and CT in relation to soft tissues (P<0.001, R^2^ = 0.85).

**Figure 2:**
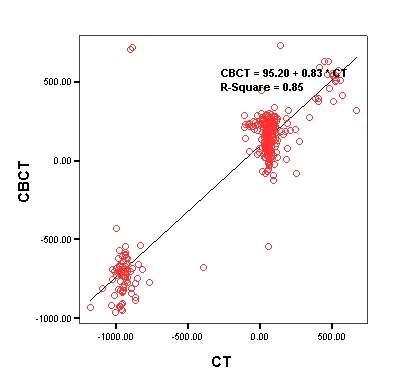
The scatter plot between CBCT and CT in relation to soft tissues.

Figure 2 presents the linear regression equation between CBCT and CT in relation to soft tissues.

**Figure 3:**
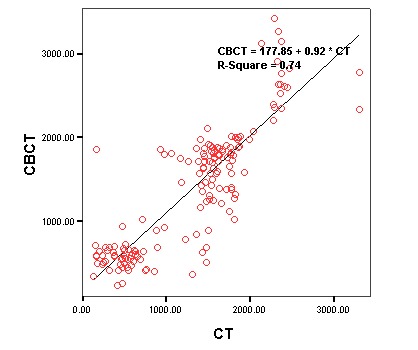
The scatter plot between CBCT and CT in general.

### Analysis of Hard Tissues

Linear regression analysis was used to evaluate the relationship between CBCT and CT about hard tissues. Table 1 presents the results of the analysis. Based on the results, there was a strong linear relationship between CBCT and CT in relation to hard tissues (P<0.001, R^2^ = 0.74). Figure 3 presents the linear regression equation between CBCT and CT concerning hard tissues.

### Overall Analysis of Tissues

**Figure 4:**
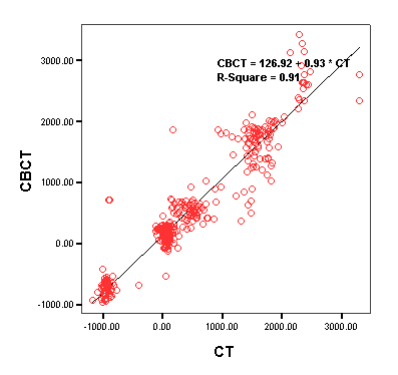
The scatter plot between CBCT and CT in relation to hard tissues.

Linear regression analysis was used to evaluate the relationship between CBCT and CT in general. The results of the analysis are presented in Table 1. Based on the results, there was a strong linear relationship in general between CBCT and CT (P<0.001, R^2^ = 0.91). Figure 4 shows the linear regression equation between CBCT and CT in general.

**Table 1:** The results of linear regression analysis for comparison of different tissues on CT and CBCT images

**Tissue type**	**P-value**	**Constant**	**B coefficient**	**R**	**R** ^ 2 ^
**Soft**	P<0.001	95.20	0.83	0.92	0.85
**Hard**	P<0.001	177.85	0.92	0.86	0.74
**Total**	P<0.001	126.92	0.93	0.95	0.91

## Discussion

Several studies have compared the HU in CT with the gray level in CBCT techniques, and favorable results have been achieved. However, due to differences in the gray level values of various CBCT units, the quantitative application of gray level in CBCT has been avoided.

Valiyaparambil et al^5^ evaluated hard-tissue equivalent materials;the results of the present study showed a weaker linear correlation in terms of the numeric values compared to the study above.However, both studies indicated a strong linear correlation between HU and gray level and the differences in the results of these two studies might be attributed to the selection of samples. In the study by Valiyaparambil et al the cortical and trabecular bone equivalents were homogeneous and due to the resin coverage of soft-tissue equivalent, the hard tissue equivalent was not affected by the heterogeneous soft tissues, while in the present study, the soft and hard tissues were heterogeneous and the hard tissue was affected by the heterogeneous soft tissue.

Similar to the study above, Mah et al^3^ carried out a study using air and different volumes of water to simulate tissues as closely as possible; the linear coefficient achieved in that study was similar to that in the present study. It appears the results of the study above can be extended to human samples because different volumes of water were used to compensate for differences in human subjects’ body sizes.

In a study by Reeves et al^8^ that followed the study above, the effect of soft tissues on hard tissues was evaluated in a more real manner by placing bite blocks in the patients’ oral cavities. Despite a strong linear correlation, the study above did not compare real HU and gray level, and gray levels from two CBCT units were compared with the HU values derived from the radiation attenuation coefficient equation.

Furthermore, some studies were carried out on the dry mandible by Parsa et al,^4^ Bujtor et al^11^ and Casseta et al,^12^ with Casseta et al using a resin template on the dry mandible to mimic soft tissues. In all the three studies, there was a strong correlation between the HU and gray level, which was higher than that in the present study.

The presence of various soft and hard tissues (tongue and the vertebral column) in the vicinity of the area evaluated might have been a confounding factor in determining the gray level, rustling in the attenuation of radiation beams reaching the target tissue. In addition, changes in the dry mandible in vitro, compared to the living human mandible, might be a factor responsible for such a difference.

A study on physiologic tissues was carried out on sheep head, in which the images produced by different CT scan units were compared.^13,14^ Despite the differences in the gray levels of different CBCT units, the differences were not statistically significant, which might be explained by the characteristics the CBCT units have in common that affect the gray level. In addition, a strong linear correlation was achieved between the HU and gray level at different kVps. In the present study, a CBCT unit with a fixed kVp was used, and since living human samples were used, metallic artifacts and motion might have been present, explaining the weaker correlation compared to the study above.

In the majority of studies, a head phantom has been used with the reconstruction of hard and soft tissues; one of the disadvantages of this technique is that the samples are not heterogeneous, while the physiologic tissues are heterogeneous.^15^

Based on a report by Plachtovics et al,^16^ a 15-unit change in the mean of the gray level due to the rotation of an asymmetrical central phantom might be detected. In addition, in vitro studies have shown that the materials surrounding an abject can affect the gray level, and based on a study by Namura et al, a significant difference has been observed with the application of water versus air as background materials.^17^

Some of the physical properties affecting the gray level are kVp, beam hardening, noise, scattered radiation and the duration of radiation.^17,19,20,21^ Some properties of the object, too, affect the gray level, including the tissue type and the homogeneity of its structure that affect the image density, finally resulting in changes in the gray level. Therefore, the results of studies on non-vital structures should be interpreted carefully.

The field of view (FOV) of the scanned area, too, affects the results. In this context, Pauwels et al^22^ used a small FOV and separately evaluated objects, reporting a weak correlation between the HU and gray level in some CBCT images. Consistent with this study, Katsumata et al^7^ reported a decrease in density variations with an increase in the object size; however, there was an increase in density variations with small object sizes. Based on a report by Molteni,^23^ the advantage of using a small FOV is a decrease in artifacts. In the present study, maximum FOV was used considering the conditions of the x-ray unit and the patients’ needs.

## Conclusion

A high degree of agreement is seen between the HU in CT and the gray level in CBCT in both hard and soft tissues. Since the gray level is an important factor in CBCT for determining the bone density before placement of implants and also for determining the bone type, the use of CBCT is recommended due to its lower radiation dose and lower cost compared to CT.

## Authors’ Contributions

TRdesigned the study, interpreted the data and wrote the major part of the article and revised it. PE edited the article. NN collected and analyzed the data and helped write the paper. SR assisted in writing, drafting and revising the article.

## Acknowledgment

The authors acknowledge the financial support provided by the Research Council of Tabriz University of Medical Sciences.

## Funding

This paper was extracted from a thesis and financially supported by the research council of the Tabriz University of Medical Sciences

## Competing Interests

There are no competing interests.

## Ethics Approval

This research was approved by the Ethics Committee of Tabriz University of Medical Sciences (The letter number is IR.TBZMED.REC.1395.248). All the data of patients were confidential.
